# Supplementary material for: Chromothripsis during telomere crisis is independent of NHEJ, and consistent with a replicative origin
Source: Genome Res. 2019 May;29(5):737–49. doi: 10.1101/gr.240705.118 (PMC6499312; doi:10.1101/gr.240705.118)
Supplement: Supplemental Material [file supp_gr.240705.118_Supplemental_file_1.zip › contigs/annotated_contigs/DB83/contig.3.DB83_length_1019_mean_cov_13.6467124632.docx]

**DB83_length_1019_mean_cov_13.6467124632**

```
ATGAAGACTTAATTCAAAACTATTGCTGAGCA|TGGTGGCTCATGCCTGTAATCCCAGCACTTTGGGAGGCCGAGGTGGGTGGATCACT
                                 >chr4:126882174-126882640 + E=5e-172
TGAGCCCAGGAGTTCGAGACTAGCCTGGGCAGCATGGTGAAACCCCGTCTCTACTAAAAATACAAAAATTAGCAGGGCGTGGTGGCAGG

CACCTGTAATCCCAGTTACTCGGGAGGCTGAGGCAGGAGAGTCACTTGAAACCAGAAGGTGGAGGTTGCAGTGAGCTGAGATCGTGCCA

TTGCACTCCAGCCGGGAAAACAGGGCAAGACTCCGTCTCAAAAACAAAAAAGACCAAAAAAAACAAACCAAACCAAACAAAAAAATATT

GCCTTTTTCGATACTACCTAAGGCCTGCCCTTCTCCCATCCTGTGCCTATAAAAGCTCCAGACTCAGCCACAATGAAGAGATACGCGAC

TTTGGGTGAAAGACCATCTTCCTGTCCCCTCTCCACTGAGAGCTGTTTTGTCAC|T|TTGGCTTGAAGGCAGGGTTTCACTGGGGACCC
                                                       >chr4:126881921-126882018 - E=2e
ACCCCTATCTGCCTAGGCATTTGTCAATCTCCTGCTACTATCACAAGCATATAGACAACGGTAT|ATGCCTACTCGGCCCACTGG|GTC
-46 p=9e-03                                                                          >c
CTCATCACCCATCAATTGTCAGCATGACCTCATTCTTCTTGGATGTGGGAAAAGATATTCTTGAGTTCGGGCACACAGAAGGCTATAAT
hr4:126882654-126883058 + E=5e-231
ACTGTGACTTTCTGCCCTCCATCATTGTAAGGCAGCTGCCCCAAGTGATGGGAAGCAGCAGCAGGGCTGAGCCGGCCACAGAGCTGTAG

CCCAGAGTCGGGGAAGGGGCTGATTAAGCTGTTAACATGCTGCTGTCTGTTGGGCTGTGGGCAGATGGACTAAAAGAGCCAATTAGCAC

ATTGTAACACCCCTTCTGGGGCTTTGAGGTCATGGACACTCTTGACTGGGGACCACCACATTTCCCTCAGGGTGACATGATTGGAGCCT

GCATAGAGCCTGCCCCTGCCAGCATTTGGAATGGTAGCTGAACTC
```
